# Colour Doppler sonography in the preoperative assessment of the vascular pedicle from the anterolateral thigh flap: proposal for a mathematical formula to predict pedicle length

**DOI:** 10.1007/s00405-018-5243-6

**Published:** 2018-12-19

**Authors:** Łukasz Łuczewski, P. Machczyński, S. Marszałek, M. Szewczyk, P. Golusiński, P. Pieńkowski, B. Szybiak, L. Weselik, E. Majchrzak, J. Hauke, W. Golusiński

**Affiliations:** 1 Department of Head and Neck Surgery, Greater Poland Cancer Centre, University of Medical Sciences Poznan, Poznan, Poland; 2 Greater Poland Cancer Center, Poznan, Poland; 3 Institute of Socio-Economic Geography and Spatial Management, Adam Mickiewicz University, Poznan, Poland

**Keywords:** Colour Doppler sonography, ALT flap, Vascular pedicle, Pedicle length

## Abstract

**Introduction:**

The anterolateral thigh flap (ALT) is one of the most commonly used grafts in head and neck reconstructive surgery. In this type of surgery, it is essential that the vascular pedicle be long enough to ensure proper vascular anastomosis. However, it is difficult to accurately estimate the pedicle length preoperatively. In this context, the current study had two aims: (1) to assess the value of colour Doppler sonography (CDS) in the preoperative assessment of the vascular pedicle and (2) to develop a mathematical model to predict the length of the vascular pedicle based on the ultrasound findings.

**Materials and methods:**

Retrospective review of patients who underwent primary surgery for head and neck cancer followed by ALT flap reconstruction at our institution from 2014 to 2018. All patients underwent CDS prior to surgical excision of the flap.

**Results:**

Preoperative CDS was useful to identify the location of the vascular perforators, to estimate the pedicle length, and to identify the vascularization variant. Using the proposed mathematical formula, the estimated minimum pedicle length and actual length agreed in 73.9% of cases, increasing to 84.1% when a 5 mm margin of error was allowed. Moreover, preoperative ultrasound accurately distinguished the two anatomical variants of the ALT vasculature in nearly all cases (97.1%).

**Conclusion:**

This study confirms the value of preoperative colour Doppler sonography for ALT flap reconstruction. The proposed mathematical model provides a highly accurate method of preoperatively assessing the length of the vascular pedicle, which may be of value in head and neck surgery.

## Introduction

Head and neck cancers are a heterogenous group of cancers, and their treatment is highly challenging. Worldwide, over 600,000 new cases are diagnosed annually. At diagnosis, most patients present extensive clinical disease [1], which typically require surgical resection followed by reconstruction with tissue grafts, also known as flaps [2, 3].

Flaps are widely used in the reconstruction of the primary site after tumour resection [4]. The anterolateral (ALT) thigh flap, first described by Song et al. in 1984 [5], is one of the most commonly used flaps in head and neck reconstructive surgery, supported by a large body of evidence. The ALT flap is most commonly used in the reconstruction of the oral cavity, tongue, and oropharynx, but can also be used to reconstruct full thickness cheek deficits [6], and soft facial and scalp tissues [7]. The ALT flap is a complex fasciomyocutaneous graft, whose blood supply comes primarily from the descending branch of the lateral femoral artery. The vascular perforators, which run between the muscles of the quadriceps, have diameters of up to 1 mm [8] and correspond directly with the vascularization of the flap [9]. There are two vascularization variants, with blood supply coming from either the descending branch of the lateral femoral artery or the oblique branch of the thigh circumflex artery. Of these two variants, the first is by far the most common. The vessels run between the straight femoral muscle of the quadriceps and their length, which ranges from 8 to 16 cm, is crucial to determining the length of the donor vascular pedicle [10]. The vascular pedicle must be long enough to ensure proper vascular anastomosis within the vessels in the neck. A wide variety of imaging methods—including manual acoustic Doppler, colour Doppler sonography (CDS), computed tomography angiography, and magnetic resonance angiography—can be used to preoperatively assess the vascular pedicle of the ALT flap. However, to date, no gold standard has yet emerged [11].

In this context, the present study had two main aims: (1) to investigate the utility of colour Doppler sonography in the preoperative assessment of the vascular pedicle of the ALT flap and (2) to develop a mathematical model to preoperatively estimate the length of the vascular pedicle based on the ultrasound findings.

## Materials and methods

This was a retrospective study of patients treated at the Head and Neck Surgery Department at our institution from 2014 to 2018. During this time period, a total of 69 reconstructive surgeries of the head and neck regions were performed using the ALT flap. All patients were assessed preoperatively by CDS.

This patient cohort included 16 women (23.2%) and 53 men (76.8%), ranging in age from 30 to 73 years (mean 56.8). Histologically, most of the tumours (57/69; 82.6%) were squamous cell cancers, followed by basal cell cancer (8 cases; 11.6%), and other (*n* = 4) histological types. In all patients, staging was determined according to the TNM classification system, as follows: T4 (*n* = 32), T3 (*n* = 29), and T2 (*n* = 7). One case was classified as stage N2 due to nodal recurrence without recurrence of the primary tumour (T0).

In 28 cases, no metastatic lymph nodes were found, while 18 and 23 patients had N1 and N2 regional staging, respectively. In all cases, the cancer was localized, with no signs of distant metastasis. Table 1 shows the characteristics of the patient cohort.

**Table 1:** Characteristics of patients (*n* = 69) who underwent ALT flap reconstruction

Primary tumour localization			Histological type			TNM staging		
Oral cavity	Skin	Other	SCC	BCC	Other	T	N	M
Number of patients						Stage (number of cases)		
39	22	8	57	8	4	T0 (1)	N0 (28)	M0 (69)
						T1 (0)	N1 (18)	M1 (0)
						T2 (7)	N2 (23)	
						T3 (29)	N3 (0)	
						T4 (32)		

*SCC* squamous cell cancer, *BCC* basal cell cancer, *T* tumour, *N* lymph nodes, *M* distant metastases

### Method

**Fig. 1:**
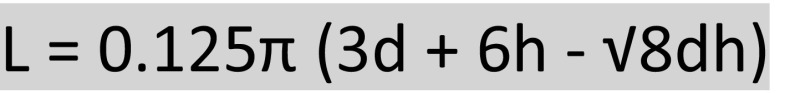
Mathematical formula to estimate for the minimum length of the vascular pedicle of the ALT flap. All measurements are in millimetres. *L* indicates the minimum length of the vascular pedicle, *d* is the distance of the perforator from the exit point of the vascular pedicle, and *h* is the maximum depth of the vascular pedicle under the quadricep muscle

Preoperative ultrasound examination of the lower limb was performed using the ALOKA SSD-3500 ultrasound (Hitachi Aloka Medical Ltd.; Tokyo, Japan) using a high-resolution linear head (7–12 MHz). CDS was used assess the blood vessels. First, the upper anterior iliac spike and lateral patellar surface were identified. Then, the centre of the line connecting these two points and the line crossing the centre at a right angle were marked. Ultrasound assessment of the vascular pedicle started at the femoral artery, applying as little pressure to the skin as possible to avoid closure of the vascular lumen. The exit point of the descending or oblique branch of the lateral femoral artery was marked on the skin of the thigh. Next, we carefully assessed the thigh centimeter by centimeter until reaching the vascular perforator. The aim of evaluating the entire course of the pedicle from the exit point from the femoral artery to the perforator is to avoid mistakes in differentiating the perforator from other vessels. The distance between the perforator marked on the skin and the point of departure of the vascular pedicle from the femoral artery (point d) was measured. The maximum depth from skin to the vascular pedicle under the quadriceps muscle (point h) was also determined. Once these coordinates have been obtained, it is then possible to estimate the minimum length of the vascular pedicle (*L*) using the proposed mathematical formula shown below (Fig. 1). Next, the estimated length of the ALT vascular pedicle was compared to the intraoperative findings. A linear regression coefficient was calculated to determine the ratio between the minimum length of the vascular pedicle estimated by ultrasound and the actual length of the surgically resected pedicle. The vascularisation variant (i.e., descending versus oblique branch) of the ALT flap was determined by ultrasound and then compared to the intraoperative findings to check for the degree of correlation.

## Results

**Fig. 2:**
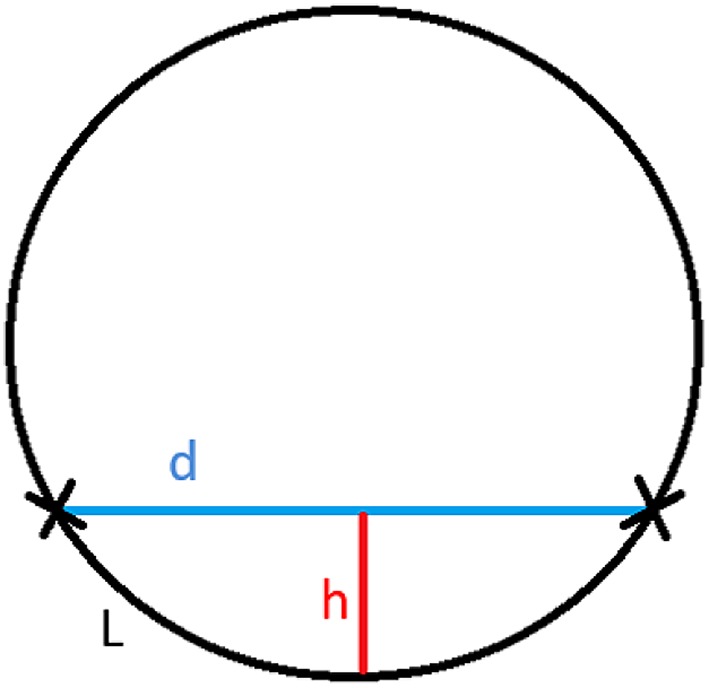
Mathematical model to assess the vascular pedicle length of the ALT flap, where *L* indicates the minimum length of the vascular pedicle (mm); *d* is the distance of the perforator from the exit point of the vascular pedicle (mm); and *h* is the maximum depth of the vascular pedicle under the quadricep muscle (mm)

In this 69 patient cohort, the vascular pedicle length estimated by ultrasound ranged from 121 to 200 mm. By comparison, the actual length of the surgically resected pedicles ranged from 120 to 200 mm. Based on the data obtained from the CDS in comparison with the intraoperative findings, we developed a mathematical formula to estimate the length of the vascular pedicle of the ALT flap (Fig. 1). This formula assumes that the mathematical model that is closest to the true shape of the vascular pedicle is a circle based on the course of the vascular pedicle that runs between the quadriceps muscles and thighs, as shown in Fig. 2.

Table 2 shows the estimated minimum length of the ALT vascular pedicle according to the mathematical formula shown in Figs. 1 and 2 for the given perforator distances (*d*) above the vascular pedicle and the maximum depth on which the vascular pedicle is located within the quadriceps femoral muscle (*h*).

**Table 2:** Estimated minimum length in mm of the ALT vascular pedicle by ultrasound

mm	**45**	**50**	**55**	**60**	**65**	**70**	**75**	**80**	**85**	**90**	**95**	**100**	**105**	**110**	**115**	**120**	**125**	**130**
*15*	***60***	***63***	***68***	***72***	***77***	***81***	***86***	***90***	***95***	***100***	***104***	***109***	***114***	***119***	***123***	***128***	***133***	***137***
*20*	***66***	***70***	***75***	***79***	***83***	***87***	***92***	***96***	***101***	***105***	***110***	***115***	***119***	***123***	***128***	***132***	***136***	***141***
*25*	***75***	***78***	***82***	***86***	***90***	***94***	***99***	***103***	***107***	***112***	***116***	***121***	***125***	***129***	***134***	***138***	***142***	***146***
*30*	***82***	***86***	***90***	***84***	***98***	***102***	***106***	***110***	***114***	***118***	***123***	***127***	***131***	***135***	***139***	***144***	***149***	***154***
*35*	***91***	***94***	***98***	***102***	***106***	***109***	***113***	***117***	***121***	***126***	***130***	***134***	***138***	***142***	***146***	***150***	***154***	***159***
*40*	***100***	***103***	***106***	***110***	***114***	***117***	***121***	***125***	***129***	***133***	***137***	***141***	***145***	***149***	***154***	***159***	***164***	***168***
*45*	***108***	***112***	***115***	***118***	***122***	***126***	***129***	***133***	***137***	***141***	***145***	***149***	***153***	***157***	***162***	***166***	***170***	***174***
*50*	***117***	***121***	***124***	***127***	***131***	***134***	***138***	***141***	***145***	***149***	***153***	***157***	***160***	***164***	***168***	***172***	***176***	***180***

Calculation are based on the mathematical formula presented in Figs. 1 and 2

The numbers given in bold represent the distance (*d*) of the perforator from the exit point of the vascular pedicle

The numbers in italic indicate the maximum depth (*h*) of the vascular pedicle under the straight thigh muscle

The minimum length of the vascular pedicle are given in bold italic

The minimum length of the ATL flap pedicle estimated according to this formula was then compared to the actual length of the surgically resected pedicle. The actual pedicle length was equal or larger than the estimated length in 73.9% of cases. This correlation increased to 84.1% when a 5 mm margin of error was allowed. The results are presented in Table 3.

**Table 3:** Correlation between minimum pedicle length estimated by ultrasound examination and the intraoperatively determined length

Comparison	Correlation (%)	Correlation (margin of error, 5 mm) (%)
Ultrasound vs. surgery	73.9	84.1

**Fig. 3:**
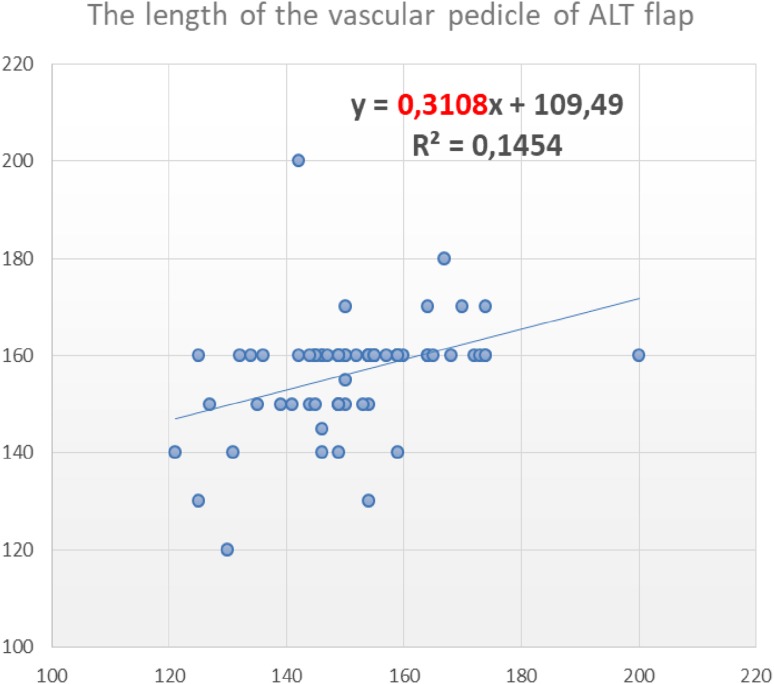
Linear regression coefficient (*R*²) expressing the ratio between the sonographically estimated length of the ALT vascular pedicle (mm) and the intraoperatively obtained length

Figure 3 shows the results of a linear regression analysis, with the relation between the predicted length and the actual length of the vascular pedicle for all 69 cases. *R*² = 0.1454 defines that the estimated and actual pedicle length agreed in 14.54% of cases.

### Anatomic variants of vascularization of the ALT flap

**Fig. 4:**
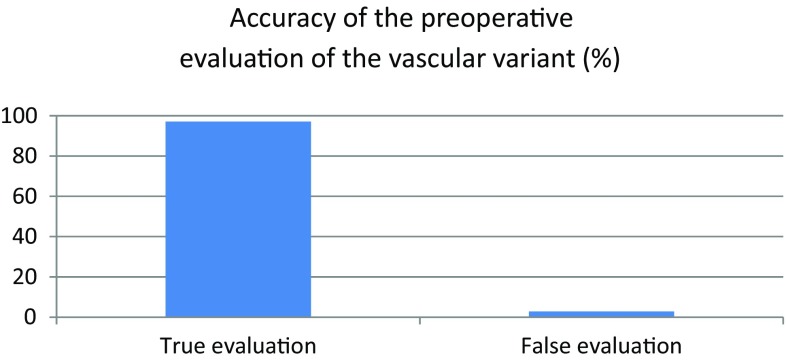
Accuracy of the preoperative ultrasound evaluation of the vascular variant of the ALT flap confirmed intraoperatively

Two anatomical variants of the ALT vasculature were identified. In most cases (67/69; 97.1%), the anatomical variant was based on the descending branch of the femoral artery. In the remaining two cases, flap vascularisation originated from the oblique branch. Figure 4 presents the correlation between the preoperative ultrasound assessment of the anatomical variant and the intraoperative assessment. The correlation was 97.1% (67/69 cases), which was highly significant (Chi-square test, *p* < 0.00001).

## Discussion

In the present study, we evaluated the utility of colour Doppler sonography to preoperatively assess the ALT flap vascular pedicle and used these sonographically obtained data to develop a mathematical formula to estimate the length of the vascular pedicle. Our finding show that CDS is an excellent tool to evaluate the pedicle. Wong et al. confirmed that there are two variants of anatomical vascularisation of the ALT lobe in their study describing the exact anatomy of the vascular pedicle of ALT [12]. In most cases, the blood supply comes from the descending branch of the femoral artery; however, less commonly, the blood supply comes from the oblique branch of the circumflex artery [13]. In the present study, most of the flaps (> 97%) were vascularised through the descending branch. Importantly, preoperative ultrasound examination correctly identified the vascularisation variant in nearly all cases (97.1%).

An important aspect of the preoperative ultrasound examination of the ALT is the accurate assessment of the vascular pedicle, particularly the length. An accurate assessment of the donor vessel is essential, because the short course of this vessel may make it difficult to obtain a vascular pedicle that is sufficiently long to properly reconstruct the site of the primary tumour excision. For this reason, it is essential to obtain detailed, accurate information on both the length of the vascular pedicle and on the anatomical variant of the ALT flap vascularisation, both of which play a role in selecting the optimal reconstructive technique [14].

The importance of accurately assessing the length of the ALT flap pedicle for proper surgical planning was demonstrated by Han et al. in their recent case report [15]. As those authors showed, careful preoperative assessment may avoid the need for later vessel grafting to lengthen the pedicle, a procedure that is both difficult and risky. Moreover, accurately determining the pedicle length preoperatively would limit the need for pedicle-lengthening surgery to emergency situations or cases in which it is not possible to use an alternative type of reconstruction.

The length of the vascular pedicle needed for reconstruction also depends on the site of the primary tumour. For example, in scalp reconstructive surgery, Lamaris et al. found that the vascular pedicle should be based on the profunda femoris artery, even when proximal neck vessels are used as the recipient targets [16]. Therefore, reconstructive operations, especially in the scalp area, require a long vascular pedicle, which is why preoperative ultrasound assessment should always be performed in such cases.

The length of the vascular pedicle depends on the location of vascular perforators, which can be determined using the mapping scheme proposed by Golusinski et al. to locate vascular perforators [17]. In most cases, the point at which the vascular pedicle is located closest to the skin is approximately at the midpoint of the thigh (field I), which explains why most vascular perforators are obtained from that field.

In our proposed mathematical formula to estimate vascular pedicle length, the distance between the perforator and this skin point (expressed in millimetres) is one of the key coordinates (point d) of the formula. The vessel runs under the quadriceps muscle, adopting an ellipsoidal shape, which is clearly seen on CDS examination. The second coordinate (*h*) in this formula is the maximum depth of the vessel under the quadriceps muscle. Based on the data obtained by the CDS, we can use the proposed formula for a section of the circle to accurately estimate the minimum length of the vascular pedicle (*L*) of an ALT flap with a high degree of accuracy.

The linear regression coefficient (*R*^2^), which expresses the ratio between the preoperatively estimated length of the ALT vascular pedicle and the intraoperatively determined length, was *R* = 0.1454, indicating that estimated and actual pedicle length agreed in 14.54% of cases. However, it is essential that the pedicle will not be shorter than the predicted length. Thus, the accuracy of the preoperative CDS assessment of the minimum pedicle length was 73.9%, increasing to 84.1% when a 5 mm margin of error was allowed, indicating that the method is reliable. Importantly, it is easy to obtain the data from CDS, without the need for additional training. As a result, this technique can be successfully applied by most clinicians in routine clinical practice.

## Study strengths and limitations

The main limitation of this study is that all ultrasound examinations were performed by a single sonographer; therefore, all measurements and the assessed minimum pedicle length may vary when performed by multiple sonographers. To our knowledge, this is the first attempt to mathematically estimate the length of the vascular pedicle, which is an important strength of this study.

## Conclusion

Colour Doppler sonography provides precise information about the donor site in the preoperative assessment of the ALT flap. The mathematical model presented in this study for assessing the minimum length of the vascular pedicle of the ALT flap allows for the accurate preoperative assessment of the pedicle length. This formula may be of value to head and neck surgeons.
